# Influence of the molecular adsorption of CO_2_, CO and NO on the stability of oxygen vacancies on the anatase TiO_2_ (101) surface

**DOI:** 10.1039/d5ra10116f

**Published:** 2026-04-28

**Authors:** Zhi-Wen Wang, Jie Zhang, Meng-Ting Yue, Wei-Guang Chen, Ming-Yu Zhao, Ya-Nan Tang

**Affiliations:** a College of Physics and Electronic Engineering, Zhengzhou Normal University Zhengzhou 450044 China zwwang@zznu.edu.cn yntang2010@163.com

## Abstract

The impact of the molecular adsorption of CO_2_, CO and NO on the stability of oxygen vacancies at the anatase TiO_2_ (101) surface is studied through first-principles calculations. Our findings reveal that the adsorption of CO_2_, CO, and NO stabilizes the surface oxygen vacancy relative to the subsurface vacancy, with total energies being 0.08 eV, 0.32 eV, and 1.58 eV lower, respectively. This suggests that the adsorption of these molecules can thermodynamically reverse the relative stability between the surface and subsurface oxygen vacancies, with the surface oxygen vacancy surface becoming the most stable. Additionally, we investigate the kinetic effects of oxygen vacancy interactions with small molecules. The diffusion barriers for oxygen vacancies on surfaces with adsorbed CO_2_ and CO are found to be 0.68 eV and 0.36 eV, respectively—significantly lower than the diffusion barriers on the clean surface by 0.16 eV and 0.50 eV, respectively. These results suggest that CO adsorption can effectively promote the diffusion of oxygen vacancies. Overall, this study highlights the crucial role of molecular adsorption in modulating the stability and interaction of oxygen vacancies on the anatase TiO_2_ (101) surface, providing insights into the photocatalytic activity of this material.

## Abstract 2

The impact of the molecular adsorption of CO_2_, CO and NO on the stability of oxygen vacancies at the anatase TiO_2_ (101) surface is studied through first-principles calculations.

## Graphical abstract

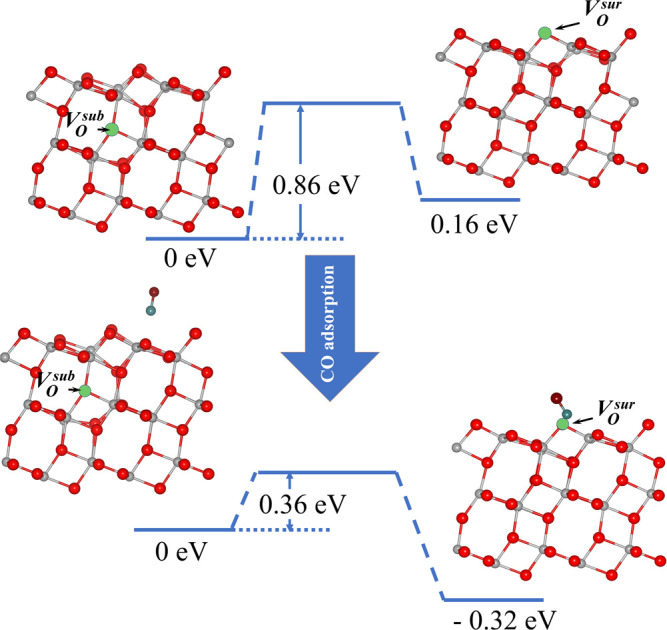

## Introduction

Titanium dioxide (TiO_2_) has been extensively studied due to its promising applications in heterogeneous catalysis, solar cells, gas sensors, photocatalysis, and environmental cleaning.^1–9^ Although rutile TiO_2_ is the thermodynamically stable phase, the (110) surface has been widely used as a model surface.^10–12^ However, the anatase phase exhibits greater stability relative to rutile when nanoparticle dimensions are reduced below 11 nm (ref. 13), while also demonstrating superior surface reactivity.^14^ Among anatase surfaces, the (101) surface is the most stable.^15^ The interactions between small molecules and the TiO_2_ (101) surface have been studied extensively in recent years.^16–19^

Surface oxygen vacancies (V^sur^_O_) are key defects on the TiO_2_ (110) surface and play a crucial role in surface reactions.^8,11,20–27^ In contrast, studies have shown that subsurface oxygen vacancies (V^sub^_O_) are more stable than surface vacancies on the anatase TiO_2_ (101) surface.^26,27^ As a result, there has been limited research on the interaction between small molecules and defective anatase (101) surfaces.^17–19^ Notably, Setvin *et al.* reported that O_2_ adsorption could reverse the relative stability of V^sur^_O_ and V^sub^_O_.^17^ Additionally, other studies have shown that water and methanol can facilitate the migration of oxygen vacancies (V_O_s) from the subsurface to the surface on the anatase (101) surface.^18,19^ We previously studied the interaction between H_2_S and oxygen vacancies on the anatase TiO_2_ (101) surface and found that H_2_S adsorption could reverse the stability of V^sur^_O_ and V^sub^_O_.^28^ Upon the adsorption of small molecules such as H_2_O, H_2_S and methanol, V_O_s on the anatase TiO_2_ (101) surface undergo a stabilization transition from the subsurface to the surface layer. These V_O_s can now function as accessible active sites, as they are no longer obstructed by steric hindrance or electronic screening from the surface molecular adlayer. Consequently, the decomposition of adsorbed small molecules including H_2_O, H_2_S, and methanol is significantly promoted, leading to an improved surface photocatalytic performance.^18,19,28^

Several important reactions occur on the anatase TiO_2_ (101) surface, such as the photocatalytic reduction of CO_2_ to methanol for fuel production,^16,29^ low-temperature CO oxidation and CO hydrogenation reactions,^30–33^ and the oxidation of NO to HNO_3_ to reduce nitrogen oxide emissions from vehicles and industrial sources.^34,35^ However, studies on these reactions typically overlook the stability of oxygen vacancies in the subsurface, and the effect of adsorbed molecules on the stability of oxygen vacancies is not well understood in such works. Additionally, the role of oxygen vacancies in various photocatalytic reactions remains unclear, which limits the practical application of anatase TiO_2_ as a catalyst.

In this study, we systematically investigate the impact of molecular adsorption (CO_2_ CO, and NO) on the stability of V_O_s at the anatase (101) surface. Our results show that after CO_2_, CO, and NO adsorption, the surface with V^sur^_O_ becomes more energetically favorable than the surface with V^sub^_O_. This suggests that the relative stability of the surface with either a V^sub^_O_ or V^sur^_O_ can be reversed, with the surface containing V^sur^_O_ becoming the most stable. We also examine the kinetics of V_O_ diffusion. We find that the diffusion barriers of V_O_s from the subsurface to the surface with CO_2_ and CO adsorption are only 0.68 eV and 0.36 eV, which are significantly lower than the clean surface by 0.16 eV and 0.50 eV, respectively. These findings indicate that CO adsorption can significantly enhance the diffusion of V_O_s.

## Computational methods

The calculations were based on density functional theory (DFT) using the Perdew–Wang 91(PW91) generalized gradient approximation^36,37^ and the Vienna *ab initio* simulation package (VASP) code with projector-augmented wave pseudopotentials.^38,39^ An energy cutoff of 500 eV was used for expanding the Kohn–Sham wave functions. The anatase TiO_2_ (101) surface was modeled as a (1 × 4) supercell slab. The stoichiometric slab containing three O–Ti–O trilayers (Ti_48_O_96_) and a vacuum with a thickness of 20 Å, with a *Γ*-centered 2 × 2 mesh, has been tested to be well converged. The positions of atoms in the bottom trilayer are fixed to mimic the bulk, and the other atoms were relaxed until the forces converged to 0.01 eV Å^−1^. To model a reduced anatase TiO_2_ (101) surface, an oxygen atom was removed from the slab; the reduced slab contained 48 Ti atoms and 95 O atoms, and the V_O_ density was 1/4, which was enough for the small molecules to get adsorbed on the surface with V_O_s.^28^ To investigate the reaction kinetics by locating the transition states, we employed the nudged elastic band (NEB) method within the VASP framework.^40^ For each elementary step, the reaction coordinate was represented by five intermediate images constructed through a linear interpolation between the boundary states. Structural relaxations along the reaction pathway were performed using the conjugate gradient scheme, with a force convergence criterion of 0.05 eV Å^−1^. The reaction barrier was defined as the energy difference between the initial state to the saddle point, identified using the image with the maximum electronic energy along the minimum energy pathway.

The adsorption energy of small molecules on the TiO_2_ (101) was calculated using the following equation:^41^

1 *E*_ads_ = *E*_tot_ − *E*_sur_ − *E*_mol_ − *E*_ZPE_ − *PV* + *TS*,

where *E*_tot_ is the total energy of a molecule adsorption on the slab, *E*_sur_ is the total energy of TiO_2_ (101) surface before small molecule adsorption, *E*_mol_ is the energy of a molecular on a 15 × 15 × 15 Å^3^ vacuum box, *E*_ZPE_ is the zero-point energy, *V* is the volume of the vacuum box, *P* and *T* is the relevant temperature and pressure of the box, respectively, and *S* is the entropy of small molecules. The chemical potentials of small molecules can be referenced to the total energy of the elementary phases at *T* = 0 K. The formation energies of the defects are always positive or the crystal would be unstable;^42^ positive (negative) energies are intended to be an endothermic (exothermic) reaction.

## Results and discussion

**Fig. 1:**
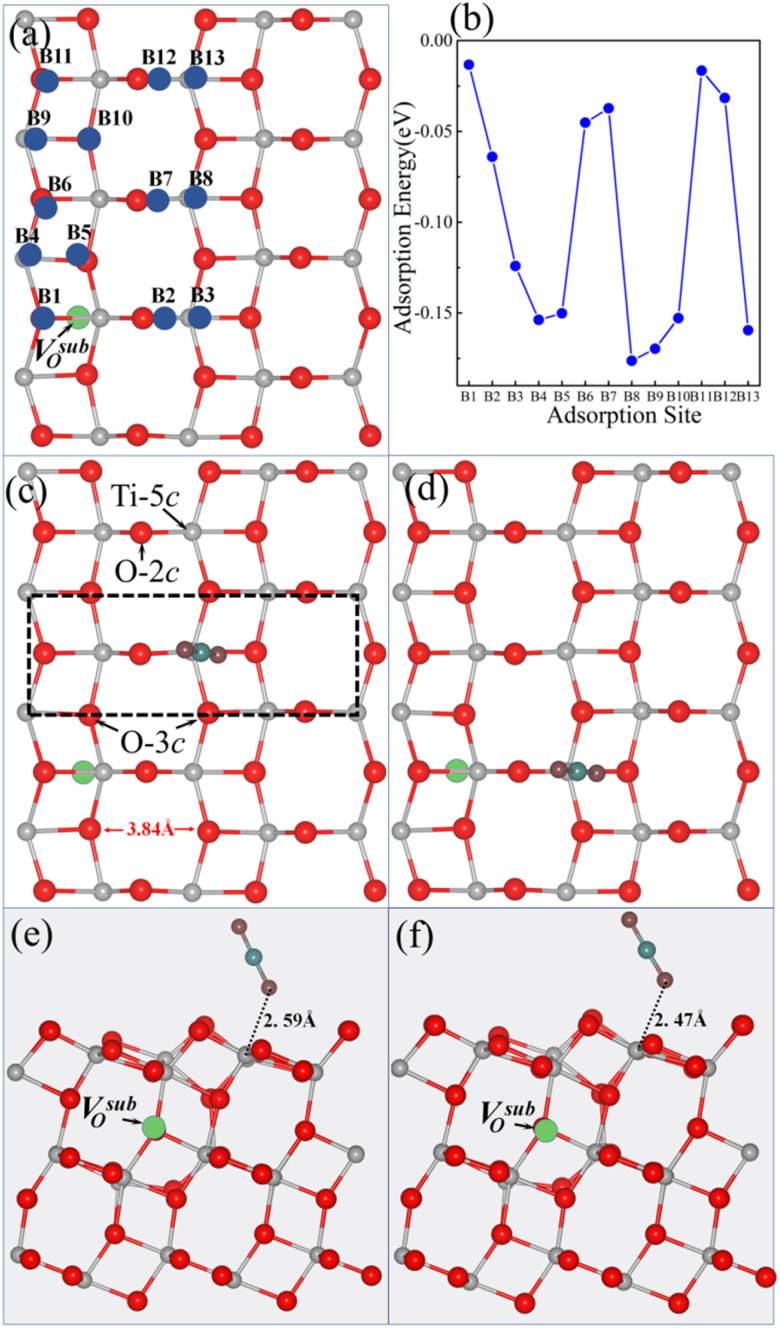
(a) Top view of a 1 × 4 supercell of the anatase TiO_2_ (101) surface with a V^sub^_O_ and thirteen possible adsorption sites for small molecules. (b) Calculated adsorption energies of CO_2_ at all the thirteen sites on the V^sub^_O_-containing surface. (c) Top and (e) side views of the optimized structure of the CO_2_-adsorbed B8 site. The black dashed square in (c) outlines the primitive cell of the anatase (101) surface. (d) Top and (f) side views of the optimized structure of the CO_2_-adsorbed B3 site. In the structural diagrams, gray, red, cyan, and burgundy spheres represent titanium (Ti), surface lattice oxygen (O), carbon (C), and oxygen atoms (O) from the CO_2_ molecule, respectively. The green dashed circles indicate the position of the V^sub^_O_.

Our previous studies showed that the anatase TiO_2_ (101) surface system containing a V^sub^_O_ is more stable than that with a V^sur^_O_ as the surface containing V^sur^_O_ is 0.16 eV higher in total energy than the surface containing V^sub^_O_.^28^ Upon introducing a V^sub^_O_ into the anatase TiO_2_ (101) surface, we considered thirteen potential adsorption sites for small molecules, as shown in Fig. 1a. The CO_2_ molecule maintains a linear geometry; in the calculations for all the 13 adsorption sites, it was placed vertically above the surface, and the vertical distance between the lower oxygen atom of the molecule and the surface atom directly beneath the adsorption site was 1.92 Å in each case. After relaxation, the adsorption energy of CO_2_ on the anatase TiO_2_ (101) surface with a V^sub^_O_ was calculated using eqn (1). As illustrated in Fig. 1b, the most stable adsorption site for CO_2_ is the B8 site, with the optimized geometry shown in Fig. 1c and e. The most stable adsorption site corresponded to the next-nearest five-coordinated titanium atom (Ti-5c) site, where the distance between the lower oxygen atom of CO_2_ and Ti-5c was 2.59 Å, the angle between CO_2_ and the surface was 63.3°, and the adsorption energy at the B8 site was −0.18 eV. Additionally, the adsorption energy of CO_2_ at the B3 site was −0.13 eV, where CO_2_ got adsorbed on the nearest Ti-5c site. The energy of this site was only 0.05 eV higher than of the B8 site. The optimized geometry for this configuration is shown in Fig. 1d and f; here, the distance between the lower oxygen atom of CO_2_ and Ti-5c was 2.47 Å, which was shorter than at the B8 site, and the angle between CO_2_ and the surface was 58.9°.

**Fig. 2:**
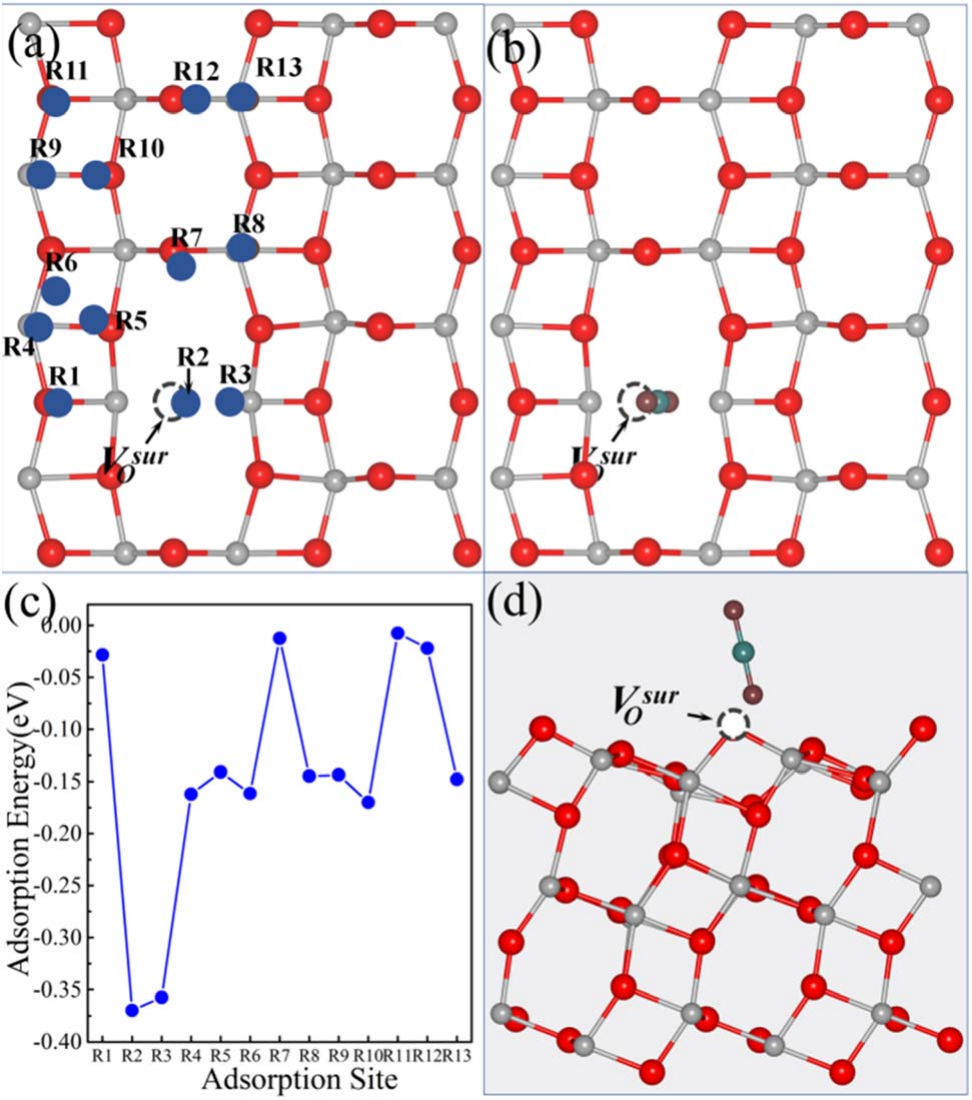
(a) Top view of the anatase TiO_2_ (101) surface with a V^sur^_O_, showing the thirteen possible molecule adsorption sites labeled R1–R13. Top view (b) and side view (d) of the optimized atomic structure of the CO_2_-adsorbed R2 site. (c) Calculated adsorption energies of CO_2_ at the thirteen different sites on the anatase (101) surface with a V^sur^_O_. In all panels, the V^sur^_O_ sites are represented by black dashed spheres. In the structural diagrams, gray, red, cyan, and burgundy spheres represent titanium (Ti), surface lattice oxygen (O), carbon (C), and oxygen atoms (O) from the CO_2_ molecule, respectively. The dashed circles indicate the position of the V^sur^_O_.

Fig. 2a illustrates the thirteen possible adsorption sites for small molecules on the anatase TiO_2_ (101) surface with a V^sur^_O_. Similarly, for CO_2_ adsorption at the 13 adsorption sites on the anatase (101) surface containing a V^sur^_O_, the initial configurational setup was identical to that used for the surface models with a V^sub^_O_. When CO_2_ was adsorbed on a surface oxygen vacancy, the distance between the bottom oxygen atom of CO_2_ and the original oxygen atom at the vacancy was 1.92 Å. The adsorption energies after relaxation are shown in Fig. 2c. Results indicated that the most stable adsorption site for CO_2_ was the R2 site, with the optimized geometric structures shown in Fig. 2b and d. The most stable adsorption occurs at the top of the V^sur^_O_, where the angle between CO_2_ and the surface is 77.6°, and the adsorption energy at the R2 site is −0.37 eV.

Our previous studies showed that a V^sub^_O_ is more stable than a V^sur^_O_ on a clean anatase TiO_2_ (101) surface, with the surface containing a V^sur^_O_ being 0.16 eV higher in total energy than the surface with a V^sub^_O_. As a result, after CO_2_ adsorption on the B3 and B8 sites, the surface with the V^sur^_O_ is 0.08 eV and 0.03 eV lower in total energy than the surface with the V^sub^_O_.

**Fig. 3:**
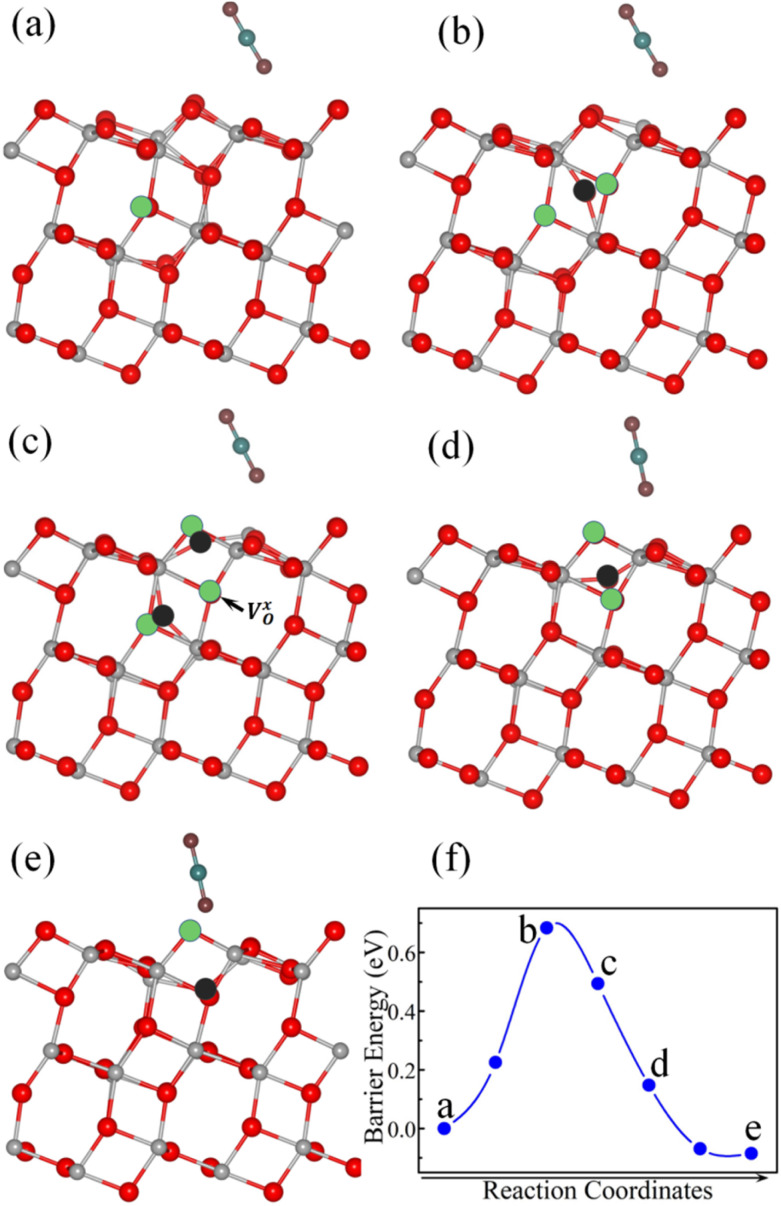
Selected optimized structures (a–e) and energy profile (f) during V_O_ diffusion from the subsurface to the surface layer with pre-adsorbed CO_2_. In the structural diagrams, gray, red, cyan, and burgundy spheres represent titanium (Ti), surface lattice oxygen (O), carbon (C), and oxygen atoms (O) from the CO_2_ molecule, respectively. The black sphere represents the specific oxygen atom undergoing migration. The green circles indicate the V_O_ sites involved in the diffusion; in panels (b)–(d), the circles are shown to highlight the migration path. All models maintain the same stoichiometry, containing only one V_O_ throughout the reaction.

After CO_2_ adsorption, the V_O_ tends to migrate from the subsurface to the surface. The calculated V_O_ diffusion pathway and optimized geometrical structures are shown in Fig. 3. Initially, the V_O_ is located in the subsurface layer, with CO_2_ adsorbed on the B3 site (Fig. 3a). The oxygen atom beneath the surface (O-2c) then diffuses to the V^sub^_O_ site (Fig. 3b) and forms a 
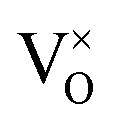
 complex (Fig. 3c). Subsequently, the surface O-2c atom diffuses to the 
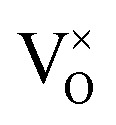
 site (Fig. 3d), forming a V^sur^_O_ with CO_2_ adsorbed at the R2 site (Fig. 3e). The energy profiles are shown in Fig. 3f. Our calculations showed that the diffusion pathway of the V_O_ from the subsurface to the surface was similar to that of the clean surface.^28^ The diffusion barrier of V_O_ on the CO_2_-adsorbed surface was 0.68 eV, which was 0.18 eV lower than on the clean surface, suggesting that CO_2_ adsorption promotes V_O_ diffusion.

**Fig. 4:**
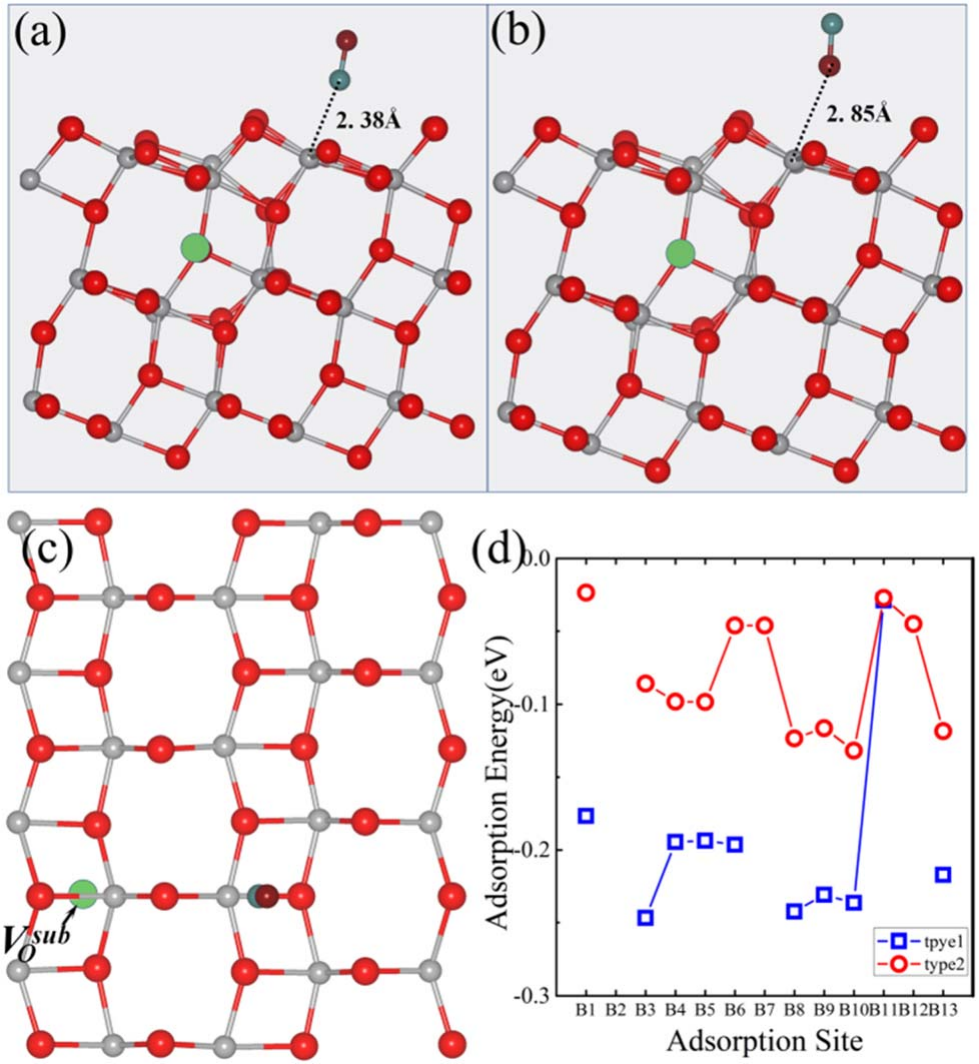
(a) Top and (c) side views of the optimized atomic structure of the CO-adsorbed B3 site on the anatase TiO_2_ (101) surface with a V^sub^_O_ in the type 1 configuration. (b) Top view of the corresponding structure in the type 2 configuration. (d) Adsorption energies of CO on the anatase (101) surface with a V^sub^_O_. The absence of data points at certain sites in (d) indicates that CO placed at these positions is unstable and spontaneously migrates to neighboring stable sites during the relaxation process. In the structural diagrams, gray, red, cyan, and burgundy spheres represent titanium (Ti), surface lattice oxygen (O), carbon (C), and oxygen atoms (O) from the CO molecule, respectively. The green dashed circles indicate the position of the V^sub^_O_.

Next, we examined the adsorption energy of CO on the anatase TiO_2_ (101) surface with a V^sub^_O_. Thirteen possible adsorption sites and two adsorption types for CO were considered. In type 1, the CO molecule was oriented vertically with the C atom closest to the surface (Fig. 4a and c), and in type 2, the CO molecule is oriented vertically with the O atom closest to the surface (Fig. 4b). The adsorption energies of CO on the anatase TiO_2_ (101) surface with a V^sub^_O_ are shown in Fig. 4d. Results indicated that CO preferred to adsorb *via* type 1 configuration, where the C atom was closest to the surface, as this configuration had a lower adsorption energy than type 2 configuration. The most stable adsorption site for CO was the B3 site (type 1), and the optimized geometric structure is shown in Fig. 4a and c. The distance between CO and Ti-5c was 2.38 Å, the angle between CO and the surface was 80.1°, and the adsorption energy was −0.25 eV. In contrast, for type 2, the distance between the O atom and Ti-5c was 2.85 Å, the angle between the CO axis and the surface was 86.5°, and the adsorption energy was −0.08 eV. Furthermore, as illustrated in Fig. 4d, the adsorption energy of type 1 is consistently lower than that of type 2 at almost all equivalent adsorption sites. This thermodynamic trend clearly indicated that the CO molecule preferentially adsorbed *via* type 1 mode, making it the most stable configuration for this system.

**Fig. 5:**
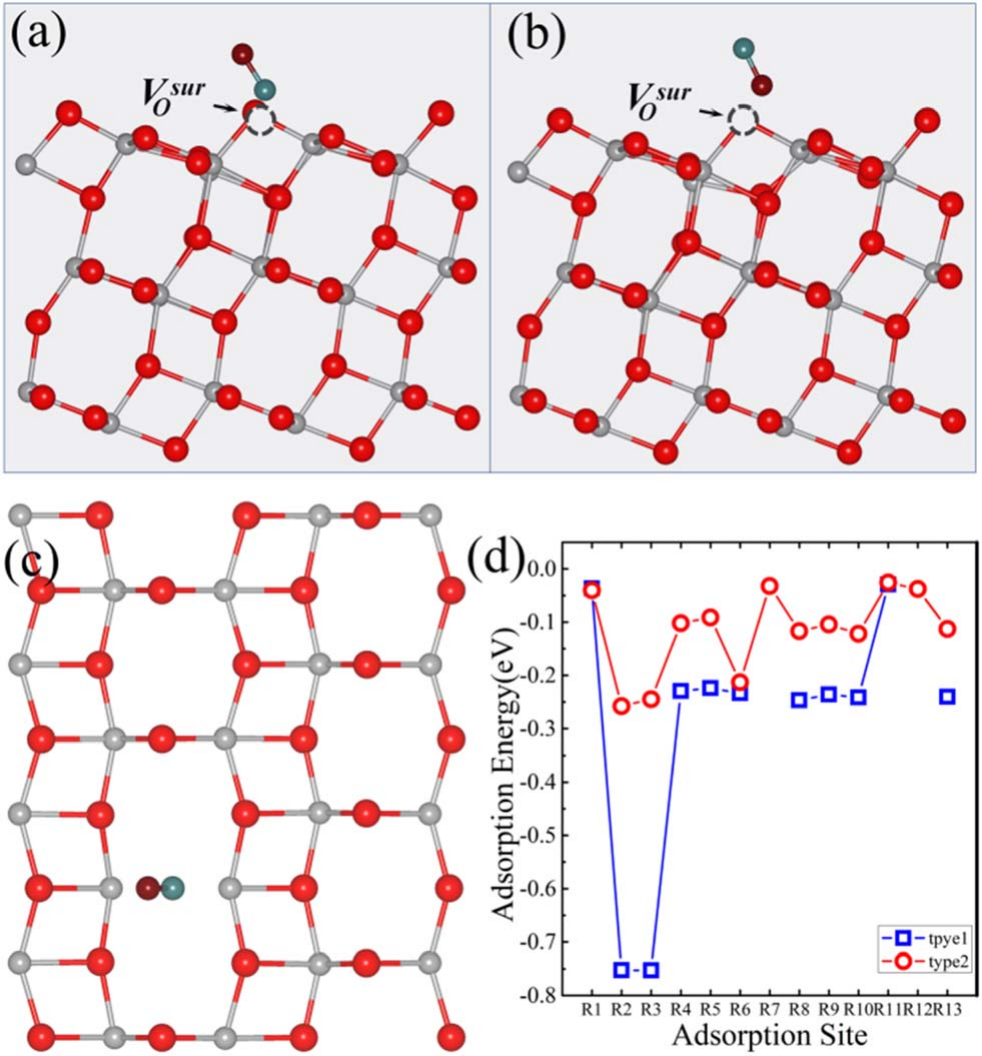
(a) Top and (c) side views of the optimized atomic structure for the CO-adsorbed R2 site on the anatase TiO_2_ (101) surface with a V^sur^_O_ in the type 1 configuration. (b) Top view of the corresponding structure in the type 2 configuration. (d) Adsorption energies of CO on the anatase (101) surface with a V^sur^_O_ for the thirteen different adsorption sites. V^sur^_O_ is represented by black dashed spheres. The absence of data points at certain sites in (d) indicates that CO placed at these positions is unstable and spontaneously migrates to neighboring stable sites during the relaxation process. In the structural diagrams, gray, red, cyan, and burgundy spheres represent titanium (Ti), surface lattice oxygen (O), carbon (C), and oxygen atoms (O) from the CO molecule, respectively. The dashed circles indicate the position of the V^sur^_O_.

We also investigated the adsorption energy of CO on the anatase TiO_2_ (101) surface with a V^sur^_O_. Thirteen potential adsorption sites (Fig. 2a) and two adsorption types for CO were considered. Similar to the surface with a V^sur^_O_, in type 1, the CO molecule was oriented vertically with the C atom closest to the surface (Fig. 5a and c), and in type 2, the CO molecule was oriented vertically with the O atom closest to the surface (Fig. 5b). The adsorption energies of CO on the anatase TiO_2_ (101) surface with a V^sur^_O_ are shown in Fig. 5d. It can be seen that, except for the R7 and R12 sites, the adsorption energies of CO in type 1 configuration were lower than those in type 2 configuration. The most stable adsorption site for CO was the R2 site in type 1 configuration, with the optimized geometry shown in Fig. 5a and c. The most stable adsorption occurred at the V^sur^_O_ site, where the angle between CO and the surface was 57.8° and the adsorption energy was −0.75 eV, which was significantly larger than the energy for the same site in type 2 configuration.

Similar to CO_2_, the surface with a V^sur^_O_ was 0.32 eV lower in total energy than the surface with a V^sub^_O_ after CO adsorption. This indicates that CO adsorption can thermodynamically reverse the relative stability of the surface with either a V^sub^_O_ or V^sur^_O_. After CO adsorption on the anatase TiO_2_ (101) surface with a V^sub^_O_, the relative stability of the configuration with the V^sub^_O_ decreased, and the surface with the V^sub^_O_ became the most stable.

**Fig. 6:**
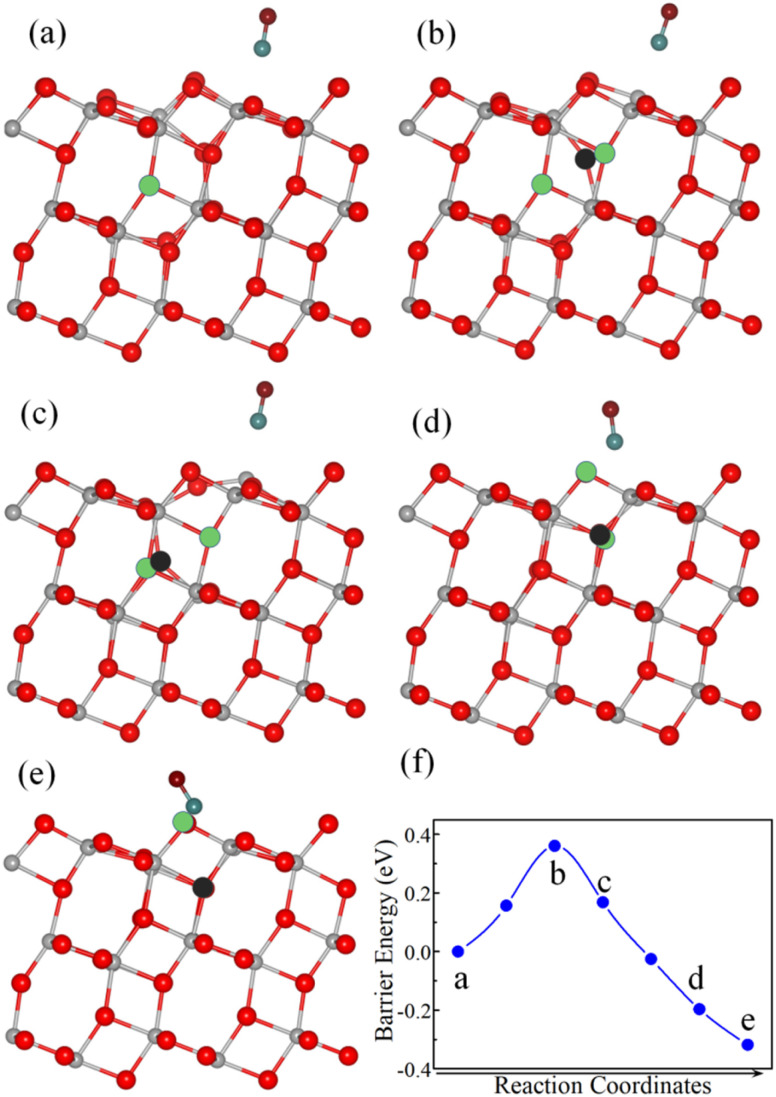
Selected optimized structures (a–e) and energy profile (f) during the V_O_ diffusion from the subsurface to the surface layer after CO adsorption. In the structural diagrams, gray and red spheres represent titanium (Ti) and surface lattice oxygen (O) atoms and the cyan and burgundy spheres represent carbon (C) and oxygen (O) atoms from the CO molecule, respectively. The black sphere represents the specific oxygen atom undergoing migration; the green circles indicate the V_O_ sites involved in the diffusion. In panels (b)–(d), the circles are shown to highlight the migration path. All models have the same stoichiometry, containing only one V_O_ throughout the process.

The diffusion of the V_O_ from the subsurface to the surface with CO adsorption is shown in Fig. 6. The diffusion pathway of the V_O_ was similar to that observed with CO_2_ adsorption. However, the diffusion barrier of the V_O_ on the surface with CO adsorption was only 0.36 eV, which was significantly lower than that on the clean surface by 0.50 eV. These results suggested that CO adsorption effectively promoted V_O_ diffusion from the subsurface to the surface layer.

**Fig. 7:**
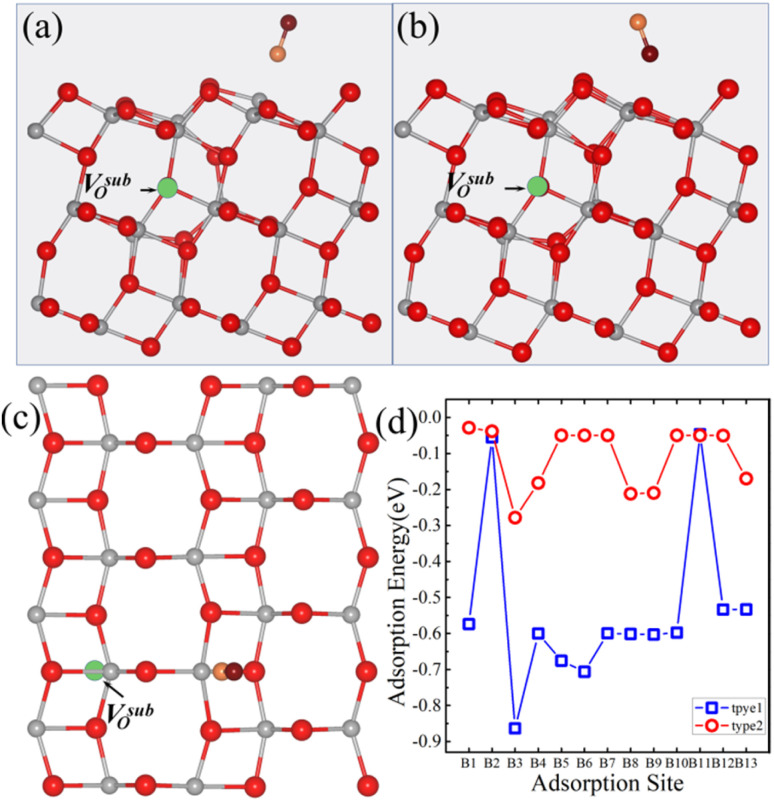
(a) Top and (c) side views of the optimized atomic structure for the NO-adsorbed B3 site on the anatase TiO_2_ (101) surface with a V^sub^_O_ in the type 1 configuration. (b) Top view of the corresponding structure in the type 2 configuration. (d) Adsorption energies of NO on the anatase (101) surface with a V^sub^_O_. In the structural diagrams, gray, red, orange and burgundy spheres represent titanium (Ti), surface lattice oxygen (O), nitrogen (N), and oxygen atoms (O) from the NO molecule, respectively. The green dashed circles indicate the position of the V^sub^_O_.

Next, we studied the adsorption energy of NO on the anatase TiO_2_ (101) surface with a V^sub^_O_. Similar to CO, thirteen possible adsorption sites and two adsorption types for NO were considered. In type 1, the N atom of NO was the closest to the surface (Fig. 7a and c), while in type 2, the O atom of NO was the closest to the surface (Fig. 7b). The adsorption energy of NO on the anatase TiO_2_ (101) surface with a V^sub^_O_ is shown in Fig. 7d. Results indicated that the B3 site was the most stable adsorption site for NO, with an adsorption energy of −0.86 eV, which was much lower than the adsorption energies of CO_2_ and CO at the same site by 0.68 eV and 0.61 eV, respectively. The B3 site was located on the nearest Ti-5c site, where the distance between the N atom of NO and Ti-5c was 1.86 Å, and the angle between NO and the surface was 70.8°.

**Fig. 8:**
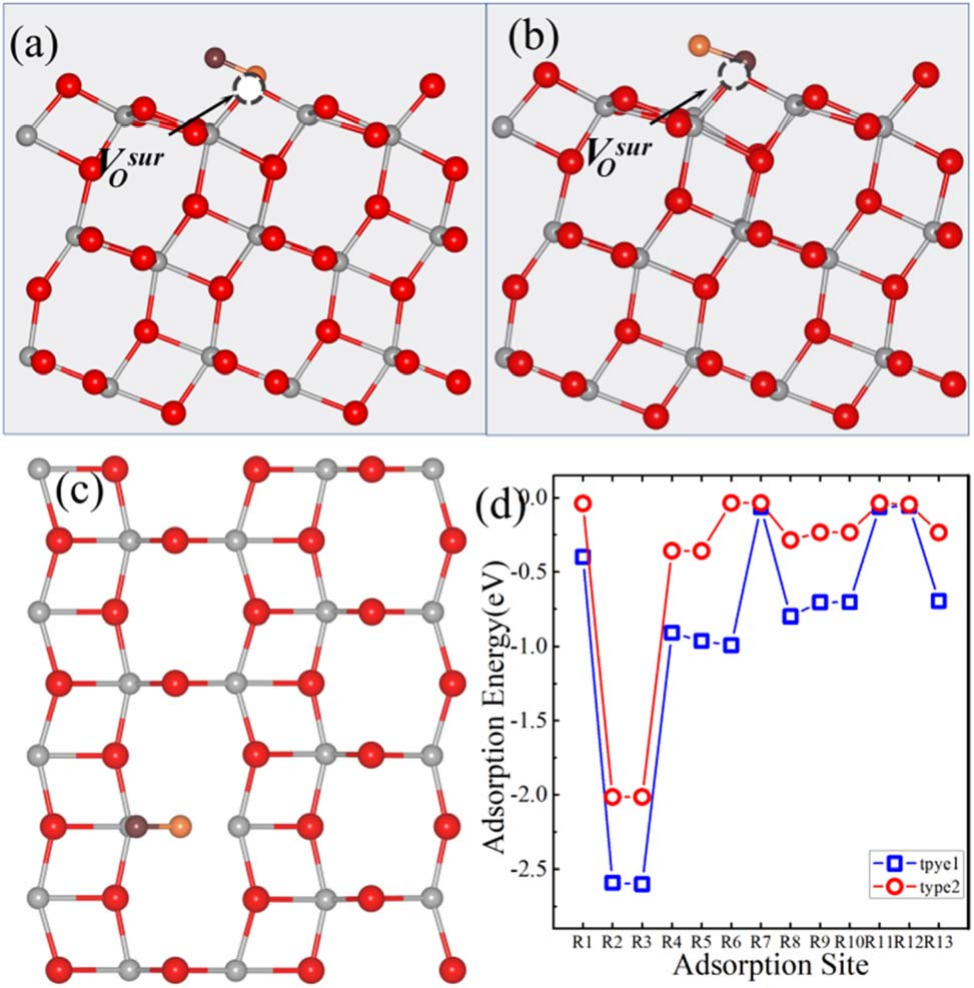
(a) Top and (c) side views of the optimized atomic structure of the NO-adsorbed R2 site on the anatase TiO_2_ (101) surface with a V^sur^_O_ in the type 1 configuration. (b) Top view of the corresponding structure in the type 2 configuration. (d) Adsorption energies of NO on the anatase (101) surface with a V^sur^_O_ for thirteen different adsorption sites. In the structural diagrams, gray, red, orange and burgundy spheres represent titanium (Ti), surface lattice oxygen (O), nitrogen (N), and oxygen atoms (O) from the NO molecule, respectively. The green dashed circles indicate the position of the V^sur^_O_.

We also studied the adsorption energy of NO on the anatase TiO_2_ (101) surface with a V^sur^_O_. The adsorption sites and the two identified configurations for NO are analogous to those observed for CO. Type 1 and type 2 configurations on the R2 sites are shown in Fig. 8(a–c). The adsorption energy of NO on the anatase TiO_2_ (101) surface with a V^sur^_O_ is shown in Fig. 8d. Results indicated that the B3 site was the most stable adsorption site for NO, with an adsorption energy of −2.60 eV, which was significantly lower than the adsorption energies of CO_2_ and CO by 2.23 eV and 1.85 eV, respectively. The N atom of NO was positioned on the V^sur^_O_ site, and the angle between NO and the surface was 24.4°, indicating that NO is positioned almost parallel to the surface.

After comparing the total energy following NO adsorption, we found that the surface with the V^sur^_O_ is 1.58 eV lower in energy than the surface with the V^sub^_O_. This suggested that the relative stability between the surfaces with the V^sub^_O_ and V^sur^_O_ can be reversed through NO adsorption.

**Fig. 9:**
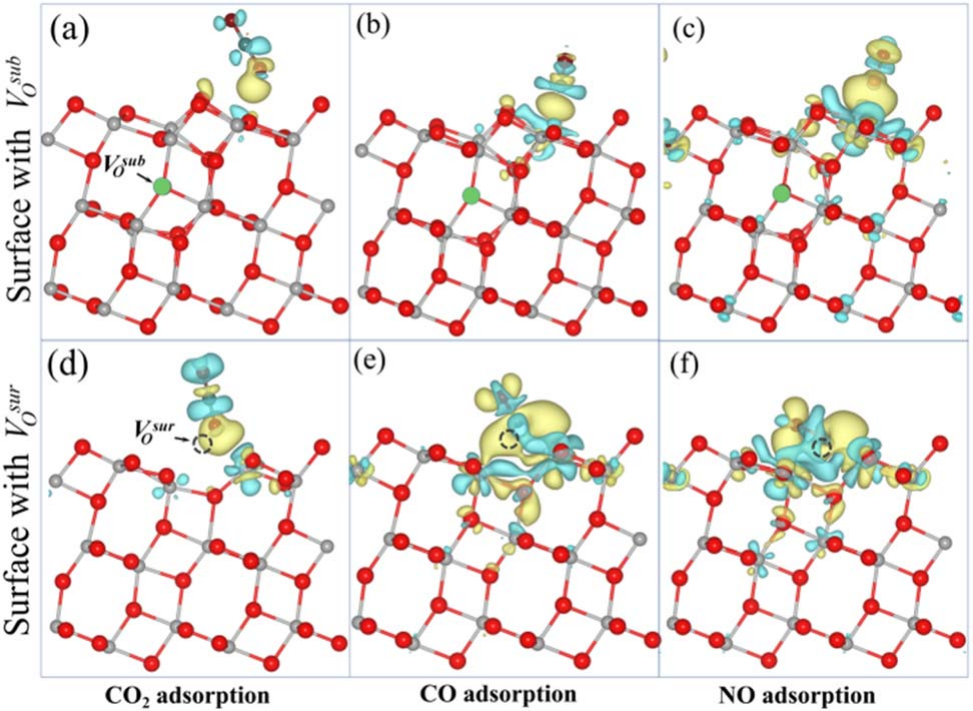
Side view of the charge density difference for CO_2_ (a and d), CO (b and e), and NO (c and f) molecules adsorbed on the anatase TiO_2_ (101) surface with a V^sub^_O_ (upper) or V^sur^_O_ (lower). The iso-surfaces represent charge accumulation (yellow) and charge depletion (cyan), providing insights into the bonding mechanisms.

To understand the adsorption characteristics of different small molecules on the anatase TiO_2_ (101) surface, we calculated the differential charge density for the stable adsorption configurations of CO_2_, CO, and NO on the anatase TiO_2_ (101) surface with a V^sub^_O_ or V^sur^_O_, as shown in Fig. 9. When CO_2_ was adsorbed on the surface with a V^sub^_O_ (Fig. 9a), the charge density around the oxygen and carbon atoms of CO_2_ decreased, while the charge density between the lowest oxygen atom of CO_2_ and the surface increased. However, the amount of charge transfer was very small, indicating the weak adsorption of CO_2_ on this surface. Conversely, when CO_2_ was adsorbed on the surface with a V^sur^_O_ (Fig. 9d), the increase in charge density between the lowest oxygen atom of CO_2_ and the surface was greater than that observed on the V^sub^_O_ surface, suggesting the stronger adsorption of CO_2_ on the surface with a V^sur^_O_. When CO was adsorbed on the surface with a V^sub^_O_ (Fig. 9b), similar to the case of CO_2_ on this surface, less charge transfer occurred. However, when CO was adsorbed on a surface with a V^sur^_O_ (Fig. 9e), the charge density around the surface atoms near the V^sur^_O_ decreased, and a significant amount of charge accumulated between CO and the surface, indicating the strong bonding between CO and this surface. When NO was adsorbed on the surface with a V^sub^_O_ (Fig. 9c), a high charge density from the vicinity of the surface atoms accumulated between NO and the surface. The amount of charge transfer was greater than that observed for CO_2_ and CO adsorption on this surface, and its adsorption energy was consequently larger. Furthermore, when NO was adsorbed on the surface with a V^sur^_O_ (Fig. 9f), the angle between NO and the surface was 24.4°, indicating that NO was positioned almost parallel to the surface. A large amount of charge was accumulated not only between the N atom of NO and the surface but also between the O atom of NO and the surface, leading to a very strong bond between NO and the surface. The close agreement between the charge analysis and adsorption energy results confirmed the robustness of our findings.

## Conclusions

We investigated the adsorption sites and energies of CO_2_, CO, and NO molecules upon their adsorption on the anatase (101) surface with a V^sub^_O_ or V^sur^_O_. On the surface with a V^sub^_O_, the most stable adsorption sites and corresponding energies are B8 (−0.18 eV), B3 (−0.25 eV), and B3 (−0.86 eV) for CO_2_, CO, and NO, respectively. On the surface with V^sur^_O_, the most stable sites and energies are R2 (−0.37 eV), R3 (−0.75 eV), and R3 (−2.60 eV) for CO_2_, CO, and NO, respectively. These charge analysis results closely match the adsorption energy findings, which validates the scientific soundness of our work. Importantly, we found that the surface with a V^sur^_O_ is 0.03 eV, 0.32 eV, and 1.58 eV lower in total energy than the surface with a V^sub^_O_ after CO_2_, CO, and NO adsorption, respectively. This indicates that small molecule adsorption can thermodynamically reverse the relative stability of the surface with either a V^sub^_O_ or V^sur^_O_. Additionally, we observed that the diffusion barriers of V_O_s on the surface with CO_2_ and CO adsorption are only 0.68 eV and 0.36 eV, respectively, which are much lower than those on the clean surface by 0.16 eV and 0.50 eV, respectively. These results suggest that CO adsorption effectively promotes V_O_ diffusion.

## Conflicts of interest

There are no conflicts of interest to declare.

## Data Availability

The data supporting the findings of this study are available within the article. Additional datasets generated and analyzed during the current study are available from the corresponding author upon reasonable request.
